# Genome-wide identification, characterization, evolutionary analysis, and expression profiling of the FCS-like zinc finger (FLZ) gene family in soybean (*Glycine max L*.) under abiotic stresses

**DOI:** 10.1038/s41598-026-67801-6

**Published:** 2026-09-08

**Authors:** Mustafa I. Abdullah, Sobhi F. Lamlom, Abdul-Hamid Emwas, Mariusz Jaremko, Nader R. Abdelsalam

**Affiliations:** 1 Agricultural Botany Department, Faculty of Agriculture, Alexandria University, Saba Basha, Alexandria, 21531 Egypt; 2 Department of Plant Production, Faculty of Agriculture Saba Basha, Alexandria University, Alexandria, Egypt; 3 Core Lab of NMR, King Abdullah University of Science and Technology (KAUST), Thuwal, 23955-6900 Makkah Saudi Arabia; 4 Smart-Health Initiative (SHI) and Red Sea Research Center (RSRC), Division of Biological and Environmental Sciences and Engineering (BESE), King Abdullah University of Science and Technology (KAUST), Thuwal, 23955-6900 Makkah Saudi Arabia

**Keywords:** *Glycine max*, FLZ domain, *GmFLZ*, Abiotic stress, Expression analysis, Genetics, Molecular biology, Plant sciences

## Abstract

Drought and salinity limit soybean yield. Despite their role in the *SnRK1* energy-sensing complex, a systematic study of FCS-Like Zinc Finger (FLZ) proteins in soybean has not been reported. We performed a genome-wide identification of the *GmFLZ* gene family, identifying 40 members distributed across 18 of the 20 soybean chromosomes. Phylogenetic analysis of 87 FLZ proteins from *Glycine max*, *Arabidopsis thaliana*, and *Oryza sativa* revealed four major evolutionary clades, suggesting that diversification predates the separation of monocots and dicots. Structural analysis identified ten conserved motifs, with Motifs 1 and 2 present in all family members. Gene duplication analysis identified 304 paralogous pairs, most arising from segmental duplication. Ka/Ks analysis indicated localized positive selection in six gene pairs and purifying selection in 97.9% of pairs. Tissue-specific expression profiling across nine tissues showed that *GmFLZ5*, *GmFLZ15*, *GmFLZ25*, and *GmFLZ34* had the highest expression levels detected across the *GmFLZ* family, with *GmFLZ5* the most highly expressed member in leaves, nodules, and stem and showing moderate expression in pod, root, and root hairs, whereas *GmFLZ18*, *GmFLZ23*, and *GmFLZ37* showed root-preferential expression. RT-qPCR validation under drought (20% PEG-6000) and salt (200 mM NaCl) treatments in the Giza 5 cultivar showed that 36 and 34 of the 40 *GmFLZ* genes, respectively, exhibited at least a two-fold change in expression, with *GmFLZ21* and *GmFLZ35* among the most strongly induced under salt stress. These findings provide an evolutionary and functional framework for the *GmFLZ* family and identify candidate genes for future functional studies in soybean stress tolerance.

**Supplementary Information:**

The online version contains supplementary material available at https://doi.org/10.1038/s41598-026-67801-6.

## Introduction

FCS-like zinc finger (FLZ) protein family, also referred to as DUF581 (domain of unknown function 581), is a plant-specific regulatory protein characterized by a highly conserved FLZ domain found in all plant lineages except algae^1–3^. FLZ proteins have been implicated in diverse biological processes, including plant growth regulation, senescence, sugar signaling, and responses to abiotic and biotic stresses^3–7^. Structurally, the FLZ domain contains approximately 70 AA residues harboring an invariant CX_2_CX_17−19_FCSX_2_C motif^5,8^. Genome-wide analyses have identified 18 FLZ genes in *Arabidopsis thaliana* (*AtFLZ*)^8^, 37 FLZ genes in maize (*ZmFLZ*)^4^, and 29 FLZ genes in rice (*OsFLZ*)^9^. FLZ proteins have also been reported to participate in protein–nucleic acid interactions^6^. Additional studies indicate that FLZ proteins function primarily through protein–protein interactions^4–6,9^. In Arabidopsis, the first FLZ domain-containing protein, mediator of ABA-regulated dormancy 1 (*MARD1*), was identified through senescence-associated enhancer trap screens and has been implicated in abscisic acid (ABA)-mediated seed dormancy, a trait with relevance to pre-harvest sprouting in grain crops^11,12^. Downregulation of *AtFLZ6* and *AtFLZ10* has been shown to enhance osmotic stress tolerance at the seedling stage, apparently via the *SnRK1* pathway^6,13^. In addition, FLZ gene members interact with SnRK (serine/threonine protein kinase) subunit^5^. *SnRK1*, for instance, functions as a regulator of sugar signaling, including sucrose metabolism and carbon allocation^14^. *SnRK2* and *SnRK3* are involved in the regulation of abiotic stress responses, including drought, salinity, and osmotic stress signaling^14,16,17^. In addition, the *ZmFLZ25* gene was upregulated by ABA through interactions with the three ABA receptors (PYL4, PYL8, and PYL12) in maize^4^. In rice, *OsFLZ18* expression was negatively regulated in plant growth under flooding stress 9. In addition to Arabidopsis, maize, and rice, studies across additional plant species, including 120 FLZ genes in wheat (*Triticum aestivum*), 56 FLZ gene in cotton (*Gossypium hirsutum*) and 19 FLZ genes in tomato (*Solanum lycopersicum*), indicate that FLZ proteins may play broad roles in regulating plant responses to environmental stress^7,18,19^. For instance, expression of the wheat FLZ gene (*TaSRHP*) enhanced tolerance to various abiotic stresses, including drought, salt, cold, and ABA 19. Also, *TaFLZ2D* is an up-regulator of salt stress tolerance and enhances tolerance to ionic pressure and ROS detoxification^7^. Moreover, four FLZs (*GhFLZ11*,* GhFLZ25*,* GhFLZ44* and *GhFLZ55*) were involved in the response to cold stress in cotton^19^. As the roles of FLZ proteins in plant growth, development, and stress responses become better understood, research attention has increasingly focused on characterizing this family to support crop improvement under abiotic stress conditions.

Worldwide demand for soybeans has been increasing to meet global population growth, which is expected to reach approximately 9.8 billion by 2050^20,21^. Soybean, considered the fourth most economically important crop after wheat, rice, and maize, provides edible oils, vegetable protein, minerals, micronutrients, fatty acids, antioxidants, vitamins, and fiber for multiple uses, as well as essential amino acids for use in food and feed^22–28^. In 2023, FAO reported that soybean cultivation covered 136.9 million hectares and resulted in a total seed production of 371.1 million tons^29^. Nevertheless, soybeans are highly exposed to various abiotic stresses, including salinity, drought, and other environmental stresses, which impact seed germination, plant growth, and development by interfering with metabolic activities, leading to yield decrease, which is expected to increase their frequency, intensity, and duration due to global climate change^28–31^.

To address these gaps, we combined bioinformatics approaches with experimental validation to conduct a genome-wide investigation of the FLZ protein family in soybean. Our objectives were to identify and characterize all *GmFLZ* gene members within the soybean genome; analyze their phylogenetic relationships, gene structures and evolutionary dynamics; predict subcellular localizations and cis-acting elements, protein–protein interaction networks; experimentally validate expression patterns under abiotic stress conditions, including salinity, drought and the interaction of salinity and drought; and identify priority candidate genes for future functional studies and crop enhancement initiatives. Together, these analyses provide a systematic framework for the FLZ gene family and may inform strategies for developing stress-tolerant soybean varieties.

## Materials and methods

### Genome-wide identification of FLZ proteins in soybean

The whole genome and proteomic data of *Glycine max* were downloaded from Phytozome v14.0 (Joint Genome Institute, JGI) (https://phytozome-next.jgi.doe.gov/; accessed on 23 January 2026) database. GmFLZ *protein* family genes were detected by an approach that Hidden Markov Model of the FLZ gene domain (PF04570) obtained from the Pfam database v38.1 (http://pfam.xfam.org/; accessed on 24 January 2026) and employed for HMM-based searches by HMMER v3.4 implemented in TBtools-II (Toolbox for biologists) v2.420^32,33^. HMM searches were conducted with an E-value threshold of 1e-5. Simultaneously, BLASTP searches used known FLZ protein sequences from *A. thaliana* and *O. sativa* as queries against the soybean proteome database, with parameters including an E-value threshold of 1e-10, a word size of 6, and the BLOSUM62 substitution matrix.

Candidate sequences from both HMM and BLAST searches were combined, and duplicate entries were removed. All candidate GmFLZ proteins were further validated using SMART v10.0 (https://smart.embl.de/) and InterProScan 108.0 (http://www.ebi.ac.uk/interpro/) to verify the presence and integrity of conserved GmFLZ domains. Only sequences containing complete GmFLZ domains (both the nucleotide-binding and substrate-binding domains) were retained for further analysis. Gene models with premature stop codons or incomplete domain structures were excluded, resulting in 40 validated *GmFLZ* genes.

### Physicochemical properties, protein sequence and subcellular localization prediction analysis

Physicochemical properties of the 40 GmFLZ proteins, including molecular weight (MW), theoretical isoelectric point (pI), grand average of hydropathicity (GRAVY), instability index, and aliphatic index, were calculated using the ProtParam tool on the ExPASy server (https://web.expasy.org/protparam/). Proteins with instability index values below 40 were considered stable. Subcellular localization was predicted with PSORT (https://wolfpsort.hgc.jp/) using plant parameters. Results were visualized as heatmaps using TBtools-II v2.420, with hierarchical clustering based on Euclidean distance and the complete-linkage method^33^.

### Sequence alignment and phylogenetic analysis of the soybean FLZ protein family

The FLZ full-length sequences of *Glycine max*,* Arabidopsis thaliana* and *Oryza sativa* were used for Multiple sequence alignments using the default parameters of ClustalW in MEGA v12.1.2. Based on the results of the sequence alignment and the neighbor-joining (NJ) method, the p-distance model was used to build a phylogenetic tree with 1000 bootstrap replicates 34. The resulting phylogenetic tree was visualized using the iTOL v7.4.2 tool (https://itol.embl.de/upload.cgi; accessed on 11 February 2026).

### Chromosomal locations and gene mapping of the FLZ protein family in soybean

The chromosomal locations of FLZ gene family members were extracted from the Glycine max genome GFF3 annotation file using TBtools-II v2.240, and the chromosomal location map of the FLZ genes was generated using TBtools-II 33.

### Gene structure and motif analysis of the FLZ protein family in soybean

Gene structure of each *GmFLZ* gene was analyzed using TBtools-II^33^ to generate gene structure diagrams showing the relative positions and sizes of exons, introns, and untranslated regions (UTRs). Furthermore, Protein Motif was conducted via the MEME tool v5.5.9 (http://meme-suite.org/tools/meme) with parameters: number of functional domains set to 10, minimum width set to 60, and maximum width set to 100 35. The XML file, the NWK file of the evolutionary tree, and the GFF file of the gene structure were processed and visualized by TBtools-II. The identified motifs were functionally annotated using the NCBI Conserved Domain Database (CDD) and Pfam database.

### Upstream cis-acting elements analysis in the promoters of *GmFLZ* members

2,000 bp upstream of promoter sequences for each *GmFLZ* gene were obtained from the Phytozome database. Cis-acting elements were predicted with PlantCARE (https://bioinformatics.psb.ugent.be/webtools/plantcare/html/), with an emphasis on stress-responsive, hormone-responsive, and tissue-specific elements. Results were visualized using TBtools II software for comparative analysis of *GmFLZ* members.

### The analysis of synteny and collinearity of *GmFLZ* members

The synteny links of *GmFLZ* genes were analyzed within the soybean genome using segmental duplications and compared with other plant species. Collinearity analysis was performed using TBtools II v 2.420 to detect paralogous and orthologous gene pairs and to display syntenic blocks. FLZ protein sequences from *Zea mays* were obtained from Phytozome v14 (https://phytozome-next.jgi.doe.gov/) using the same conserved domain criteria (Pfam: PF04570, zf-FLZ domain) as applied to *A. thaliana* and *Oryza sativa* sequences and were incorporated into the multi-species synteny analysis.

### Gene duplication analysis and evolutionary rate calculation

Gene duplication events were identified using MCScanX v1.0 implemented in TBtools-II v2.240. Duplicate gene pairs were classified as tandem duplicates if located within 100 kb on the same chromosome, or as segmental duplicates if located on different chromosomes or > 100 kb apart on the same chromosome. For each duplicate gene pair, synonymous substitution rate (Ks) and non-synonymous substitution rate (Ka) were calculated using TBtools-II. The Ka/Ks ratio was calculated to determine the type of selective pressure: Ka/Ks < 1 indicates purifying selection, Ka/Ks = 1 indicates neutral evolution, and Ka/Ks > 1 indicates positive selection. Moreover, the divergence time of duplicate gene pairs was calculated using the formula: T = Ks/(2λ). The synonymous mutation rate (λ) for soybeans is 6.1 × 10^− 9^ substitutions per synonymous site per year. So, we calculated the divergence time (T) as T = Ks/(2 × 6.1 × 10^− 9^) × 10^− 6^ Mya 36.

### Three-dimensional protein structure prediction

Representative GmFLZ protein structures were predicted using homology modeling through the SWISS-MODEL server (https://swissmodel.expasy.org/). The best templates were selected based on sequence identity and coverage. Model quality was assessed using GMQE (Global Model Quality Estimation) and QMEAN scoring functions.

### Expression patterns analysis of *GmFLZ* genes

According to transcriptome data of nine tissue types, including flower, leaves, nodules, pod, root, root Hairs, seed, shoot apical meristem, and stem, using available RNA-seq datasets. The RNA-Seq Atlas of the *Glycine max* was searched and downloaded for each soybean *FLZ* member from the phytozome v14.0 (Joint Genome Institute, JGI) (https://phytozome-next.jgi.doe.gov/). The expression data were normalized, and the heat map was visualized using Tbtools-II.

### Plant materials, stress treatments, and quantitative real-time PCR validation

Soybean seedlings (cultivar Giza 5, a commercial soybean cultivar obtained from the Agricultural Research Center, Egypt) were grown in plastic pots containing a soil-sand mixture (3:1, v/v) under controlled greenhouse conditions with a 16 h light/8 h dark photoperiod at 25 °C. At the V2 growth stage, uniform seedlings were selected and irrigated with either 200 mM NaCl or 20% (w/v) PEG-6000 solution to impose salt and drought stress, respectively. Untreated seedlings irrigated with water served as the control (0 h). Leaf tissue was collected at 1, 3, 6, 12, and 24 h after treatment, with three biological replicates per time point. Samples were immediately frozen in liquid nitrogen and stored at -80 °C until RNA extraction. Total RNA was extracted from leaf tissue using the EasyPure^®^ Universal Plant Total RNA Kit (TransGen, China) according to the manufacturer’s instructions. RNA concentration and purity were assessed spectrophotometrically (A260/A280 ratio), and integrity was verified by 1% agarose gel electrophoresis. First-strand cDNA was synthesized from total RNA using HiScript IV All-in-One Ultra RT SuperMix (Vazyme, China) according to the manufacturer’s protocol. Gene-specific primers for the 40 *GmFLZ* members were designed using Primer3, targeting amplicon lengths of 100–200 bp with melting temperatures near 60 °C, and are listed in Table S1. The soybean *Actin11* gene (*Glyma.18G290800*) served as the internal reference gene for normalization. RT-qPCR was conducted on a QuantStudio™ 1 Real-Time PCR Instrument (Applied Biosystems) using SupRealQ Ultra Hunter SYBR qPCR Master Mix (Vazyme, China), with each biological replicate run in three technical replicates. The thermal cycling program consisted of an initial denaturation at 95 °C for 3 min, followed by 40 cycles of 95 °C for 10 s and 60 °C for 30 s, with a melt-curve analysis performed after amplification to confirm primer specificity and rule out non-specific products or primer dimers. Relative expression levels were calculated using the 2^-ΔΔCt method, with fold change expressed relative to the 0 h control for each treatment. Genes were considered stress-responsive when the absolute log2(fold-change) exceeded 1 (at least a twofold change relative to the control).

### Gene enrichment analysis of the *GmFLZ* co-expression network

Gene Ontology (GO) enrichment analysis was performed on the gene set co-expressed with the *GmFLZ* family, identified from the GM-CX database as described above. Enrichment significance was assessed against genome-wide background frequencies for each GO term, with terms classified as overrepresented or underrepresented based on the ratio of observed to expected gene counts, and statistical significance evaluated after correction for multiple testing.

### Statistical analysis and data visualization

All statistical analyses were performed using R software v4.2.0. Correlation analyses were conducted using the Pearson correlation coefficient. Heat maps were generated using the pheatmap package with hierarchical clustering. Statistical significance was set at *P* < 0.05 for all analyses. All figures and visualizations were created using TBtools-II v2.420, R software v4.2.0 with the ggplot2 package, and Adobe Illustrator 2023. Color schemes were selected to be colorblind-friendly and publication-ready.

## Results

### Identification of the soybean FLZ gene family members

To examine copy-number variation in the FLZ gene family across the soybean genome, searches were performed using the protein domain PF04570; a total of 40 FLZ-encoding proteins were identified. After validation through domain analysis with SMART and InterPro databases, all 40 candidates had complete and intact GmFLZ domains, confirming their status as genuine GmFLZ family members. The soybean FLZ genes were named *GmFLZ*1-*GmFLZ40* based on the chromosomal locations of the 40 proteins. GmFLZ13 is a 105-amino-acid (aa) protein, indicating a relatively short structure, whereas GmFLZ7 is a notably longer protein at 443 aa. The protein molecular weight ranged from 11.97 (GmFLZ13) to 49.02 (GmFLZ7) kDa, and the theoretical PI ranged from 5.72 (GmFLZ27) to 10.09 (GmFLZ24), indicating moderate variation in physicochemical properties among *GmFLZ* members. Furthermore, the grand average of hydropathicity (GRAVY) values ranged from − 1.076 to -0.225, and 39 of 40 contained two exons, except for *GmFLZ7* (three exons). Furthermore, the Instability index analysis indicated that 39 of 40 GmFLZ proteins were predicted to be unstable (instability index > 40), suggesting that most GmFLZ family members may exhibit flexible structural features characteristic of regulatory proteins. Moreover, the aliphatic index ranged from 35.66 to 88.19, indicating variable thermostability among family members. Almost one-third of GmFLZ members exhibited a high aliphatic index (> 70). These results suggest that, despite high predicted instability indices, a subset of GmFLZ members may retain moderate-to-high thermostability (Table S2).

### Chromosomal location and genomic distribution of *GmFLZ* genes

**Fig. 1:**
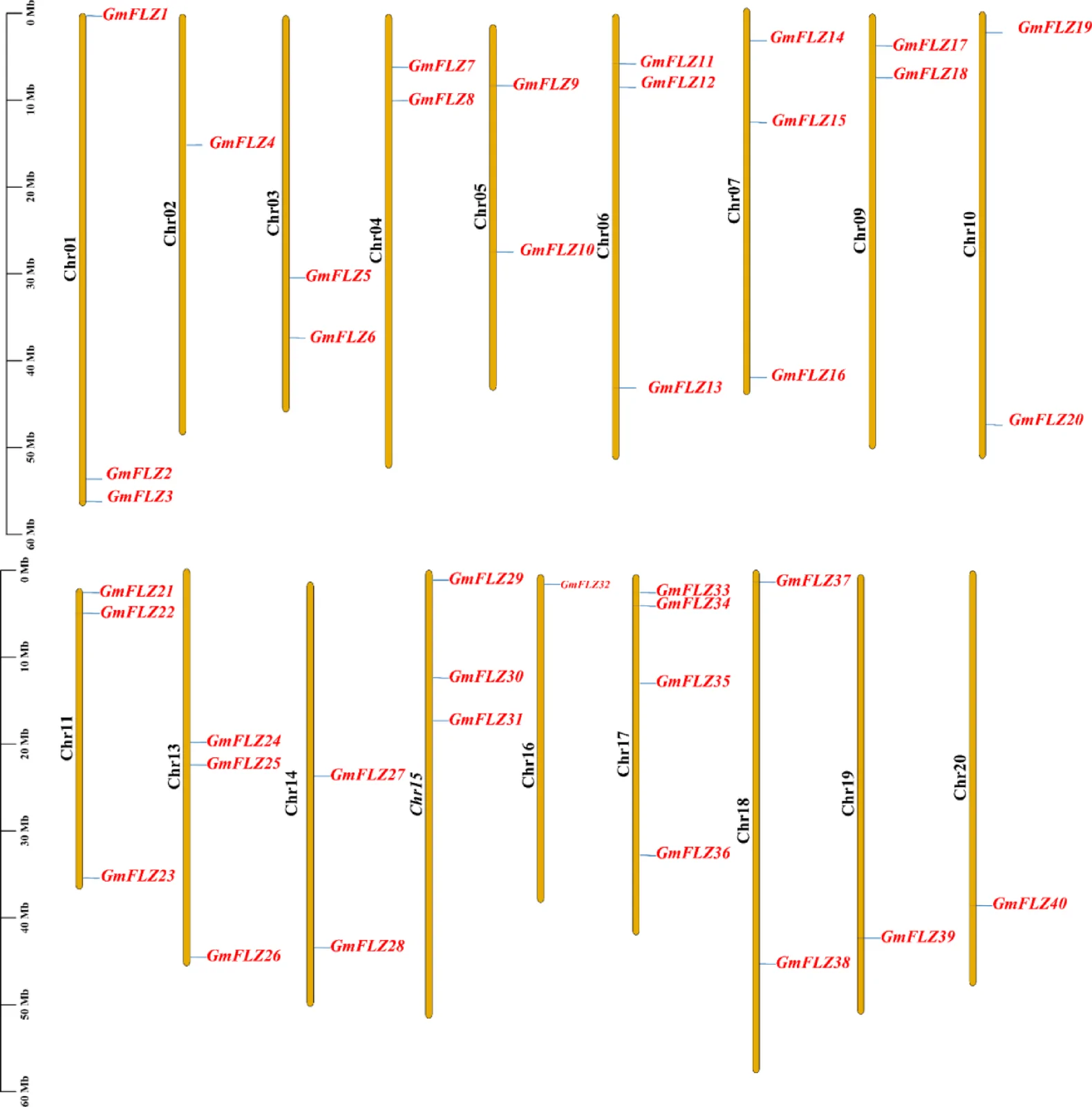
Chromosomal distribution of the *G. max* FLZ gene family.

To understand the genomic distribution of soybean *FLZ* genes across chromosomes, we examined their chromosomal locations. The 40 *GmFLZ* genes are unevenly distributed on 18 of the 20 chromosomes of *the G. max genome*, with no *GmFLZ* genes identified on chromosomes 8 and 12. The highest number of genes is found on chromosome 17 (four genes: *GmFLZ33*,* GmFLZ34*,* GmFLZ35*, and *GmFLZ36*). Three *GmFLZ* genes are located on each of chromosomes 1, 6, 7, 11, 13, and 15. Two *GmFLZ* gene members were detected on each of chromosomes 3, 4, 5, 9, 10, 14, and 18. while chromosomes 2, 16, 19, and 20 contained only a single *GmFLZ* gene (Fig. 1).

### Phylogenetic and structural analysis of soybean FLZ protein family

**Fig. 2:**
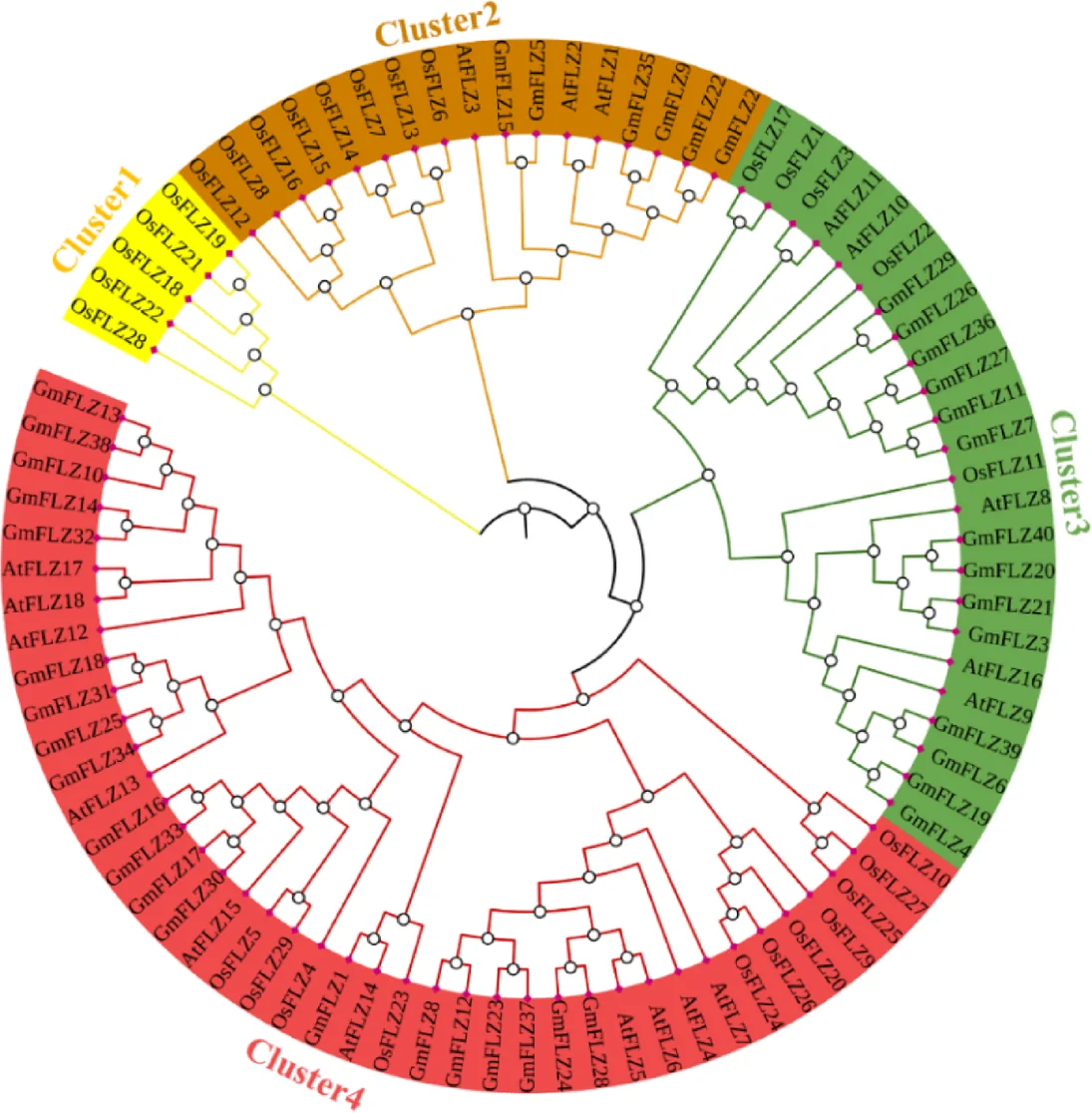
Phylogenetic tree of full-length FLZ protein sequences from soybean, Arabidopsis, and rice. The 40 FLZ in soybean, 18 FLZ in *Arabidopsis*, and 29 FLZ in rice were aligned by ClustalW, and the phylogenetic tree was constructed using MEGA12 software by the Neighbor-Joining (NJ) method. The Bootstrap value was 1000 replicates. Each cluster (1–4) is shown *via* a specific color.

To further elucidate the phylogenetic relationships among FLZ gene members in 40 *Glycine max*, 18 *Arabidopsis thaliana*, and 29 *Oryza sativa*, a phylogenetic tree was constructed from full-length protein sequences using the Neighbor-joining method with 1000 bootstrap replicates. As shown in Fig. 2 and Table S3, 87 FLZ proteins (40 GmFLZs, 18 AtFLZs, and 29 OsFLZs) are naturally divided into 4 major clades, designated cluster1, cluster2, cluster3, and cluster4, consisting of 5, 17, 24, and 41 FLZ gene members, respectively. The smallest subfamily (cluster1) contains only rice FLZ genes, whereas the other three clades contain FLZ members from soybean, rice, and Arabidopsis (Fig. 2). Several FLZ members tend to cluster in species-specific clades. Notably, cluster 2 contains 8 *OsFLZ* and 6 *GmFLZ* members, whereas only 3 *AtFLZ* genes were placed. Furthermore, cluster 3 comprises 14 *GmFLZ* members. The distribution trends of FLZ members in *A. thaliana* and *O. sativa* were similar, with a significant presence in cluster 3, which included 5 *AtFLZ* and 5 *OsFLZ* genes. Additionally, cluster4 had the largest number of *GmFLZ* members (20), *OsFLZ* members (11), and *AtFLZ* genes (10). All FLZ members within each clade share a common ancestor, suggesting that the diversity of plant FLZ proteins developed before the evolutionary split between monocots and dicots (Fig. 2).

### Subcellular localization predictions analysis of soybean FLZ genes

**Fig. 3:**
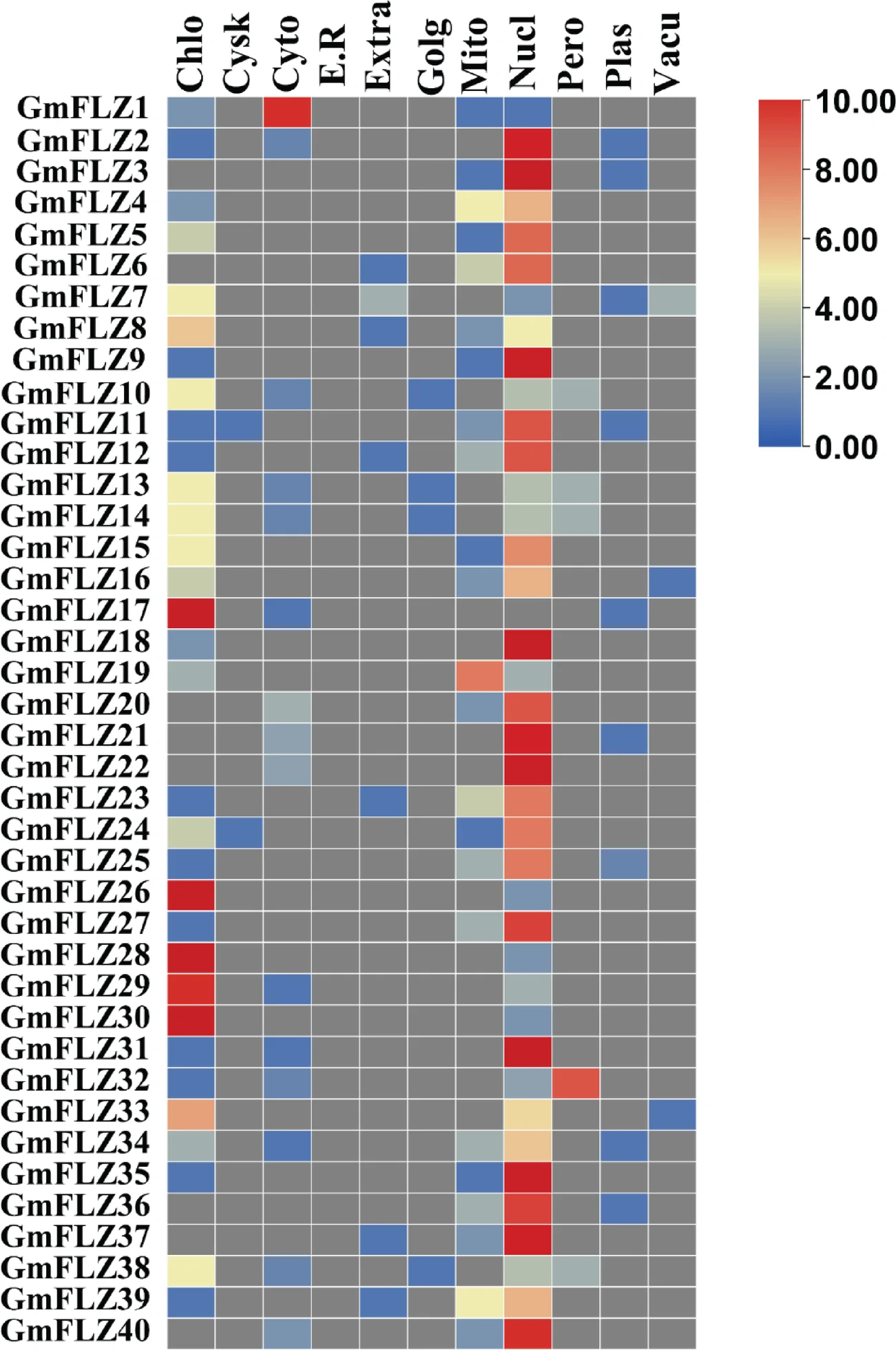
Subcellular localization prediction and expression patterns of GmFLZ proteins. The heatmap displays the predicted subcellular localization scores for 40 GmFLZ proteins across 11 different cellular compartments. Rows represent individual GmFLZ proteins (GmFLZ1 to GmFLZ40), while columns indicate different subcellular compartments: chloroplast (Chlo), cytoskeleton (Cysk), cytoplasm (Cyto), endoplasmic reticulum (E.R), extracellular (Extra), Golgi apparatus (Golg), mitochondria (Mito), nucleus (Nucl), peroxisome (Pero), plasma membrane (Plas) and vacuole (Vacu). Color intensity represents WoLF PSORT prediction scores, with red indicating high confidence scores (10), blue indicating low scores (0) and intermediate colors (yellow to orange) representing moderate confidence levels. Gray cells indicate neutral or baseline prediction values.

Subcellular localization prediction analysis was conducted for 40 FLZ proteins using PSORT (https://wolfpsort.hgc.jp/) with plant-specific parameters to determine their predicted intracellular targeting and distribution patterns (Fig. 3 and Table S4). The analysis evaluated the likelihood of protein localization across 11 different subcellular compartments, including chloroplast (Chlo), cytoskeleton (Cysk), cytoplasm (Cyto), endoplasmic reticulum (E.R), extracellular (Extra), Golgi apparatus (Golg), mitochondria (Mito), nucleus (Nucl), peroxisome (Pero), plasma membrane (Plas), and vacuole (Vacu). The prediction scores, ranging from 0 to 10, were visualized as a heatmap using hierarchical clustering based on Euclidean distance and the complete-linkage method, revealing diverse subcellular localizations that reflect the functional specialization of GmFLZ proteins across various cellular processes. The hierarchical clustering analysis revealed distinct localization patterns across the GmFLZ protein family. Several proteins showed high prediction scores for specific compartments, suggesting relatively confident subcellular targeting. For instance, *G*mFLZ2, GmFLZ3, GmFLZ9, GmFLZ18, GmFLZ21, GmFLZ22, GmFLZ31, GmFLZ35, GmFLZ37 and GmFLZ40 showed strong nuclear localization predictions, while several proteins including GmFLZ4, GmFLZ5, GmFLZ6, GmFLZ8, GmFLZ11, GmFLZ12, GmFLZ15, GmFLZ16, GmFLZ20, GmFLZ23, GmFLZ24, GmFLZ25, GmFLZ27, GmFLZ33, GmFLZ34, GmFLZ36 and GmFLZ39 showed moderate expression scores in nuclear localization. In addition, GmFLZ17, GmFLZ26, GmFLZ28, GmFL29 and GmFLZ30 exhibited the highest prediction scores for chloroplast (Chlo) localization, suggesting a possible role in chloroplast-associated stress responses. Whereas GmFLZ7, GmFLZ8, GmFLZ10, GmFLZ13, GmFLZ14, GmFLZ15, GmFLZ33 and GmFLZ38 showed intermediate expression level in chloroplast prediction. Only GmFLZ1 showed strong cytoplasm (Cyto) targeting prediction. This finding indicates that GmFLZ proteins play a functional role in general protein folding, the cytoplasmic response and the maintenance of cellular protein homeostasis under several abiotic stresses. On the other hand, all other GmFLZ protein members showed low cytoplasmic (Cyto) predictions. Furthermore, mitochondrial localization was predicted at a moderate level for some proteins, such as GmFLZ4, GmFLZ19, and GmFLZ39, suggesting a possible role in mitochondrial protein regulation. All the GmFLZ members showed a downregulated level in the peroxisome (Pero) prediction, except GmFLZ32, which showed moderate levels, indicated by orange coloration. Moreover, low localization predictions were detected in cytoskeleton (Cysk), endoplasmic reticulum (E.R), extracellular (Extra), Golgi apparatus (Golg), plasma membrane (Plas) and vacuole (Vacu) for all GmFLZ protein members.

### Phylogenetic relationships, conserved domains, gene structures, and motif organization of the *GmFLZ* gene family

**Fig. 4:**
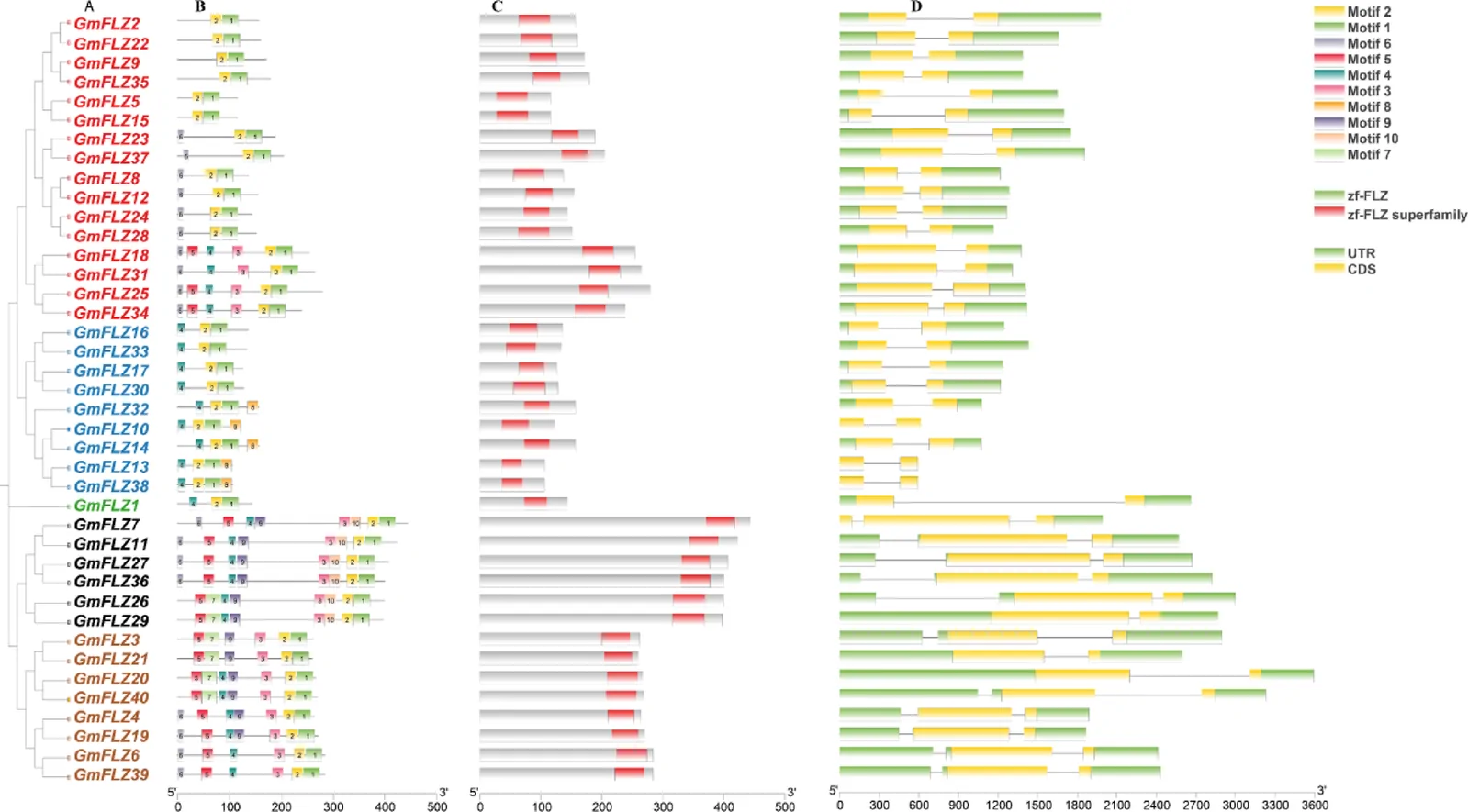
Phylogenetic relationships, conserved protein domains, gene structures, and conserved motifs of the *GmFLZ* gene family. (**A**) Neighbor-joining phylogenetic tree of the 40 GmFLZ proteins with the distribution of conserved motifs identified by MEME. (**B**) Conserved protein domain architecture showing the locations of the zf-FLZ and zf-FLZ superfamily domains. (**C**) Exon–intron organization of the *GmFLZ* genes, including coding sequences (CDS) and untranslated regions (UTRs). (**D**) Legends indicating the color codes for the ten conserved motifs, protein domains, CDS, and UTRs. The combined analysis demonstrates the close relationship between phylogenetic classification, conserved domain architecture, gene structure, and motif composition within the soybean FLZ gene family.

To investigate the evolutionary relationships and structural diversification of the soybean *GmFLZ* gene family, we conducted a comparative analysis that integrated phylogenetic classification, conserved protein domain architecture, exon–intron organization, and conserved motif composition (Fig. 4).

The phylogenetic analysis classified the 40 GmFLZ proteins into three major evolutionary groups, each exhibiting characteristic structural organization and motif composition (Fig. 4A). Members belonging to the same phylogenetic clade generally displayed highly similar conserved motif arrangements and gene structures, indicating that closely related genes have retained similar evolutionary characteristics following duplication events. Analysis of conserved protein domains revealed that all GmFLZ proteins contained the characteristic zf-FLZ domain and the corresponding zf-FLZ superfamily domain, consistent with their classification as FLZ family members (Fig. 4B). The FLZ domain was consistently positioned near the C-terminal region of most proteins, whereas the overall protein lengths varied considerably among family members, reflecting structural diversification outside the conserved functional domain.

Conserved motif analysis identified ten distinct motifs (Motifs 1–10) distributed across the soybean FLZ proteins (Fig. 4A, D). Among these, Motifs 1 and 2 were detected in all family members, indicating that they constitute the highly conserved core of the FLZ protein family. In contrast, the remaining motifs exhibited clade-specific distributions. Several members, including *GmFLZ2*,* GmFLZ5*,* GmFLZ9*,* GmFLZ15*,* GmFLZ22*,* and GmFLZ35*, possessed only the two core motifs, whereas other proteins contained additional motifs that generated more complex architectures. For example, *GmFLZ18*,* GmFLZ25*,* GmFLZ31*, and *GmFLZ34* possessed Motifs 1, 2, 3, 4, and 6, while members of the third phylogenetic group, including GmFLZ7, GmFLZ11, GmFLZ26, *GmFLZ27*,* GmFLZ29*, and *GmFLZ36*, exhibited the most elaborate motif composition containing up to seven conserved motifs. These lineage-specific motif combinations suggest progressive functional diversification following gene duplication. Gene structure analysis revealed substantial variation in genomic organization among the *GmFLZ* genes (Fig. 4C). Gene lengths ranged from less than 600 bp to approximately 3.6 kb. Most family members contained two coding exons separated by a single intron, whereas several genes displayed more complex exon–intron organizations with longer genomic regions. In contrast, *GmFLZ10*,* GmFLZ13*, and *GmFLZ38* exhibited compact gene structures with relatively short genomic lengths. Despite differences in gene size, phylogenetically related genes generally shared highly similar exon–intron structures, suggesting strong structural conservation within each evolutionary lineage. Comparison of phylogenetic relationships, motif composition, conserved domains, and gene organization demonstrated a high degree of correspondence among these four characteristics. Structurally conserved within the same phylogenetic branch, these motifs exhibited similar patterns and exon–intron organizations, indicating that structural conservation has largely been maintained during the evolution of the soybean FLZ family. Conversely, proteins with additional lineage-specific motifs and more complex gene structures likely underwent functional divergence following duplication events. Collectively, these results indicate that while the conserved *zf-FLZ* domain has been maintained throughout the evolution of the family, diversification of auxiliary motifs and gene architecture has likely contributed to the functional specialization of *GmFLZ* genes in soybean.

### Synteny and evolutionary relationships of the *GmFLZ* gene family

**Fig. 5:**
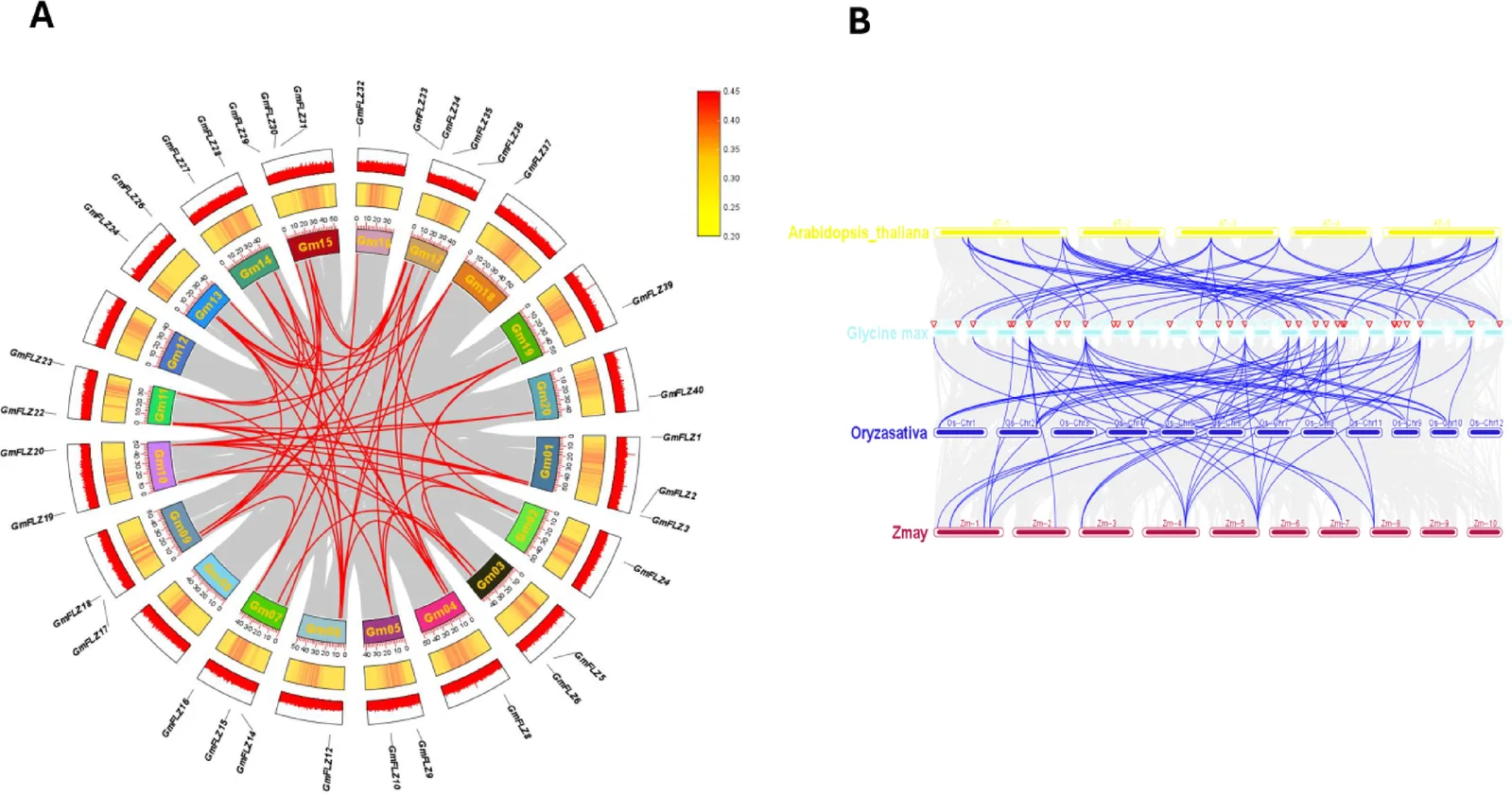
Synteny analysis and evolutionary relationships of the *GmFLZ* gene family in soybean and representative plant species. (**A**) Chromosomal distribution and intraspecies syntenic relationships of the 40 *GmFLZ* genes in the soybean (*Glycine max*) genome. Gray lines represent genome-wide collinear blocks, whereas colored connecting lines indicate duplicated *GmFLZ* gene pairs generated through segmental or whole-genome duplication events. The color scale represents chromosome density across the soybean genome, with darker colors indicating regions of higher gene density. (**B**) Comparative syntenic relationships of *FLZ* genes between soybean and three representative plant species, *Arabidopsis thaliana*, *Oryza sativa*, and *Zea mays*. Gray lines indicate genome-wide collinear blocks, while colored lines highlight orthologous *FLZ* gene pairs between soybean and each species. The conserved syntenic relationships demonstrate the evolutionary conservation of the *FLZ* gene family and suggest that its e.

To investigate the evolutionary origin and expansion of the soybean FLZ gene family, synteny analyses were performed within the soybean genome and between soybean and three representative plant species, *Arabidopsis thaliana*, *Oryza sativa*, and *Zea mays* (Fig. 5). In addition, all orthologous gene pairs identified through comparative collinearity analysis are listed in Table S7. The intraspecies collinearity analysis revealed numerous duplicated *GmFLZ* gene pairs distributed across different soybean chromosomes (Fig. 5A), indicating that the expansion of the GmFLZ family primarily resulted from segmental duplication associated with ancient whole-genome duplication (WGD) events, rather than tandem duplication. The duplicated genes were located on different chromosomes while maintaining strong collinear relationships, reflecting the paleopolyploid origin of the soybean genome.

Comparative synteny analysis demonstrated extensive conservation of FLZ genes among soybean and the three representative plant species (Fig. 5B; Table S7). A total of 47 orthologous pairs were identified between soybean and *Arabidopsis thaliana*, representing the highest degree of collinearity among the examined species. Several soybean genes, including *GmFLZ4* (*Glyma.02G146100*), *GmFLZ6* (*Glyma.03G155800*), *GmFLZ8* (*Glyma.04G104800*), *GmFLZ12* (*Glyma.06G105800*), *GmFLZ23* (*Glyma.11G238700*), *GmFLZ24* (*Glyma.13G087300*), *GmFLZ28* (*Glyma.14G170100*), and *GmFLZ37* (*Glyma.18G018800*) exhibited multiple orthologous relationships with Arabidopsis FLZ genes, suggesting that these genes have remained highly conserved during dicot evolution. Comparisons with monocot species identified numerous conserved orthologous relationships despite their greater evolutionary distance. Approximately 31 orthologous gene pairs were detected between soybean and rice (*O. sativa*), and approximately 25 between soybean and maize (*Z. mays*) (Table S7). Several soybean genes, particularly *GmFLZ8*, *GmFLZ12*,* GmFLZ23*,* GmFLZ24*,* GmFLZ28*, and *GmFLZ37*, displayed multiple orthologous counterparts in both rice and maize, indicating that these genes represent highly conserved members of the FLZ family across angiosperms. Interestingly, several soybean genes corresponded to multiple orthologous loci in the reference genomes. For example, *GmFLZ8 s*howed collinear relationships with five maize genes and four rice genes, whereas *GmFLZ23* and *GmFLZ37* each exhibited orthologous relationships with multiple genes in both monocot species. Such one-to-many orthologous relationships are consistent with the retention of duplicated FLZ genes following soybean-specific whole-genome duplication events. Overall, the extensive collinearity observed among soybean, Arabidopsis, rice, and maize indicates that the FLZ gene family originated prior to the divergence of monocotyledonous and dicotyledonous plants and has remained highly conserved throughout plant evolution. The predominance of segmentally duplicated genes, together with the retention of numerous orthologous loci, suggests that whole-genome duplication has been the primary driving force behind the expansion and evolutionary diversification of the soybean *GmFLZ* gene family.

### Cis-acting element analysis of the *GmFLZ* gene promoters

**Fig. 6:**
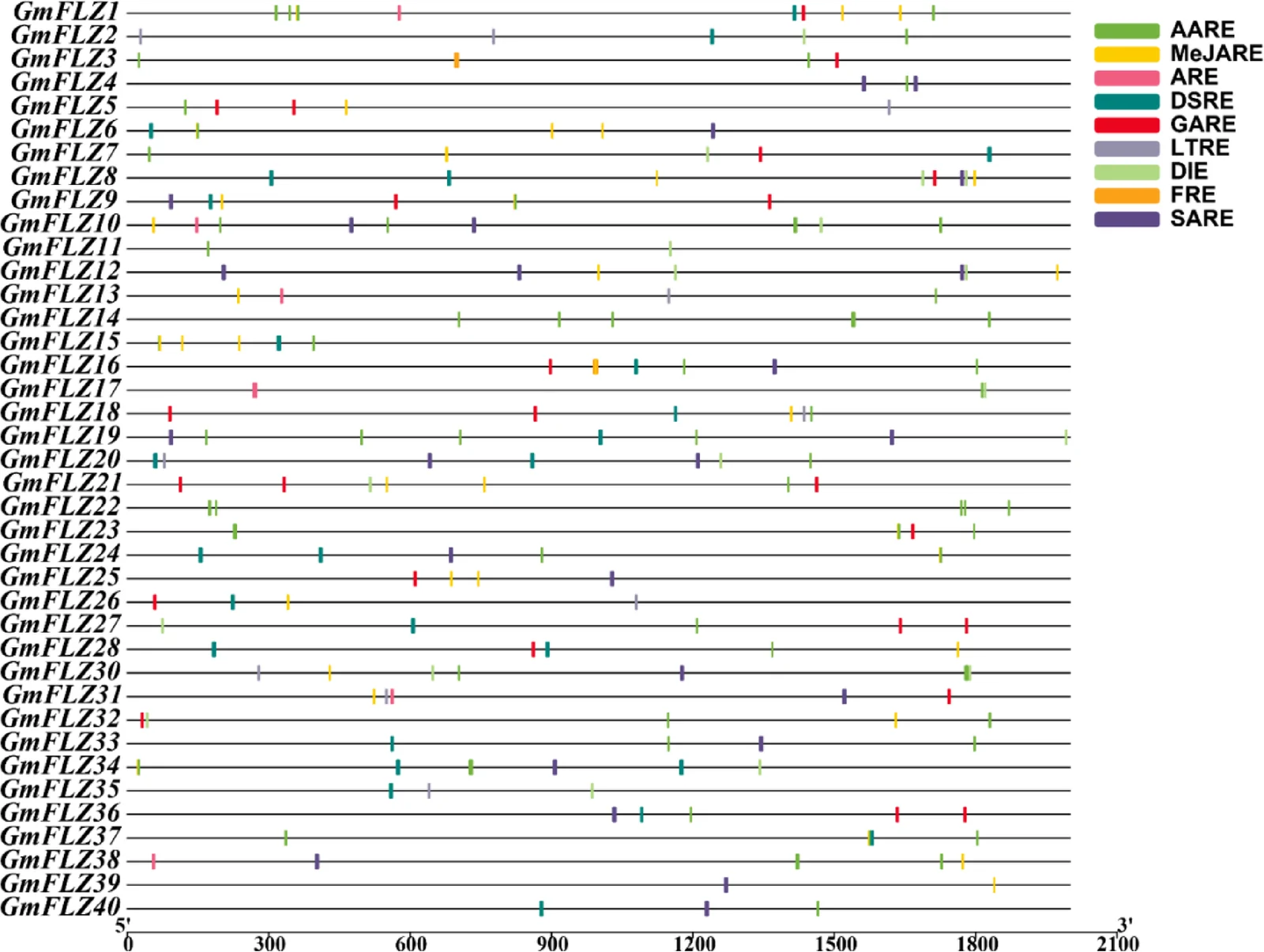
Cis-regulatory element distribution in *GmFLZ* gene promoters. Distribution patterns of 9 functional categories of cis-regulatory elements identified within 2,000 bp upstream regions of 40 *GmFLZ* genes. Each horizontal line represents a gene, with colored boxes indicating the positions and types of regulatory elements. The analysis reveals significant regulatory heterogeneity, with elements grouped into hormone-responsive (ARE; auxin, GARE; gibberellin, AARE; abscisic acid, FRE; flavonoid, SARE; salicylic acid), DSRE; defense/stress -responsive (MeJARE; MeJA responsiveness elements, LTRE; low-temperature, DIE; drought stress). The complex regulatory architecture indicates functional specialization and coordinated transcriptional control in response to environmental and developmental signals.

To gain insight into potential regulatory mechanisms governing *GmFLZ* gene expression, the 2-kb upstream promoter sequences of all 40 *GmFLZ* genes were analyzed for the presence of cis-acting regulatory elements using the PlantCARE database (Fig. 6). A diverse array of cis-elements associated with plant growth and development, phytohormone responses, and environmental stress signaling was identified, indicating that *GmFLZ* genes are likely regulated through multiple signaling pathways. A large proportion of the identified cis-elements were associated with abiotic stress responses. Among these, elements involved in drought responsiveness (MBS), low-temperature responsiveness (LTR), anaerobic induction (ARE), and stress responsiveness (TC-rich repeats) were widely distributed among the promoter regions of the *GmFLZ* genes. In addition, several promoters contained WUN-motif elements associated with wound-responsive regulation and GC-motif elements implicated in anoxic-specific inducibility. The widespread occurrence of these stress-responsive cis-elements suggests that *GmFLZ* genes may participate in multiple environmental adaptation processes. Numerous phytohormone-responsive cis-elements were also detected, indicating that the expression of GmFLZ genes is closely linked to hormone-mediated signaling pathways. Promoters frequently contained ABRE elements involved in abscisic acid (ABA) responsiveness, CGTCA-motif and TGACG-motif associated with methyl jasmonate (MeJA) signaling, TCA-element related to salicylic acid (SA) responsiveness, TGA-element and AuxRR-core involved in auxin responses, GARE-motif, P-box, and TATC-box associated with gibberellin (GA) responsiveness, and the ERE element responsive to ethylene. Among these hormone-related elements, ABRE and the MeJA-responsive motifs were the most abundant, suggesting that ABA- and jasmonate-mediated pathways may play prominent roles in regulating *GmFLZ* gene expression during stress adaptation. In addition to stress- and hormone-related elements, several cis-regulatory motifs associated with plant growth and development were identified. These included the CAT-box, involved in meristem expression; the O2-site, related to zein metabolism regulation; the RY-element, associated with seed-specific regulation; and circadian regulatory elements implicated in circadian rhythm control. Furthermore, a high frequency of light-responsive elements, including Box 4, G-box, GT1-motif, ACE, AE-box, and TCT-motif, were observed across promoter regions, suggesting that light signaling may contribute to transcriptional regulation of the *GmFLZ* family.

Considerable variation in both the number and composition of cis-elements was observed among individual *GmFLZ* promoters. Some genes possessed promoters enriched with multiple stress- and hormone-responsive elements, whereas others contained relatively fewer regulatory motifs. This diversity suggests functional divergence in the transcriptional regulation of individual *GmFLZ* genes and may explain their differential expression patterns across tissues and in response to environmental stimuli. Overall, the promoter analysis revealed that the *GmFLZ* gene family contains a rich repertoire of cis-acting regulatory elements associated with abiotic stress responses, phytohormone signaling, and developmental processes. The abundance of ABA-, MeJA-, drought-, and low-temperature-responsive elements provides strong evidence that *GmFLZ* genes are likely integrated into complex regulatory networks controlling soybean growth, development, and adaptation to adverse environmental conditions. These findings are consistent with subsequent expression analyses showing that several *GmFLZ* genes exhibit stress-responsive transcriptional patterns under drought and salt stress.

### Gene duplication analysis and evolutionary rates

To investigate the evolutionary mechanisms underlying the expansion of the soybean FLZ gene family, duplicated gene pairs were identified and their evolutionary rates were evaluated by calculating the synonymous substitution rate (Ks), nonsynonymous substitution rate (Ka), and Ka/Ks ratio (Supplementary Table S8). A total of 304 duplicated gene pairs were identified among the *GmFLZ* genes, including 290 segmentally duplicated (SD) gene pairs and 14 tandemly duplicated (TD) gene pairs, indicating that both duplication mechanisms contributed to the expansion of the soybean FLZ gene family, with segmental duplication representing the predominant evolutionary force. The calculated Ka/Ks ratios varied considerably among duplicated gene pairs, ranging from 0.00 to 2.63 (Supplementary Table S8). Notably, 298 of the 304 duplicated gene pairs (98.0%) exhibited Ka/Ks values below 1.0, indicating that the vast majority of *GmFLZ* genes have evolved under purifying (negative) selection, which has maintained the conservation of protein-coding sequences following duplication. In contrast, only six gene pairs showed Ka/Ks ratios greater than 1.0, suggesting that these genes may have experienced positive selection, potentially contributing to functional divergence during soybean evolution.

The estimated duplication times, calculated from Ks values, ranged from approximately 2.9 to 264.0 million years ago (Mya), indicating that the *GmFLZ* gene family has undergone multiple rounds of duplication throughout soybean evolutionary history. The broad distribution of duplication times suggests that both ancient and relatively recent duplication events contributed to the expansion of this gene family.

Overall, the predominance of segmentally duplicated genes together with the widespread occurrence of Ka/Ks < 1 indicates that whole-genome duplication and segmental duplication have been the primary drivers of *GmFLZ* family expansion, while strong purifying selection has preserved the structural integrity of most duplicated genes. The small number of gene pairs showing evidence of positive selection may represent candidates for functional diversification and adaptive evolution within the soybean FLZ gene family.

### The expression patterns of soybean *GmFLZ* candidate genes in 9 tissues

**Fig. 7:**
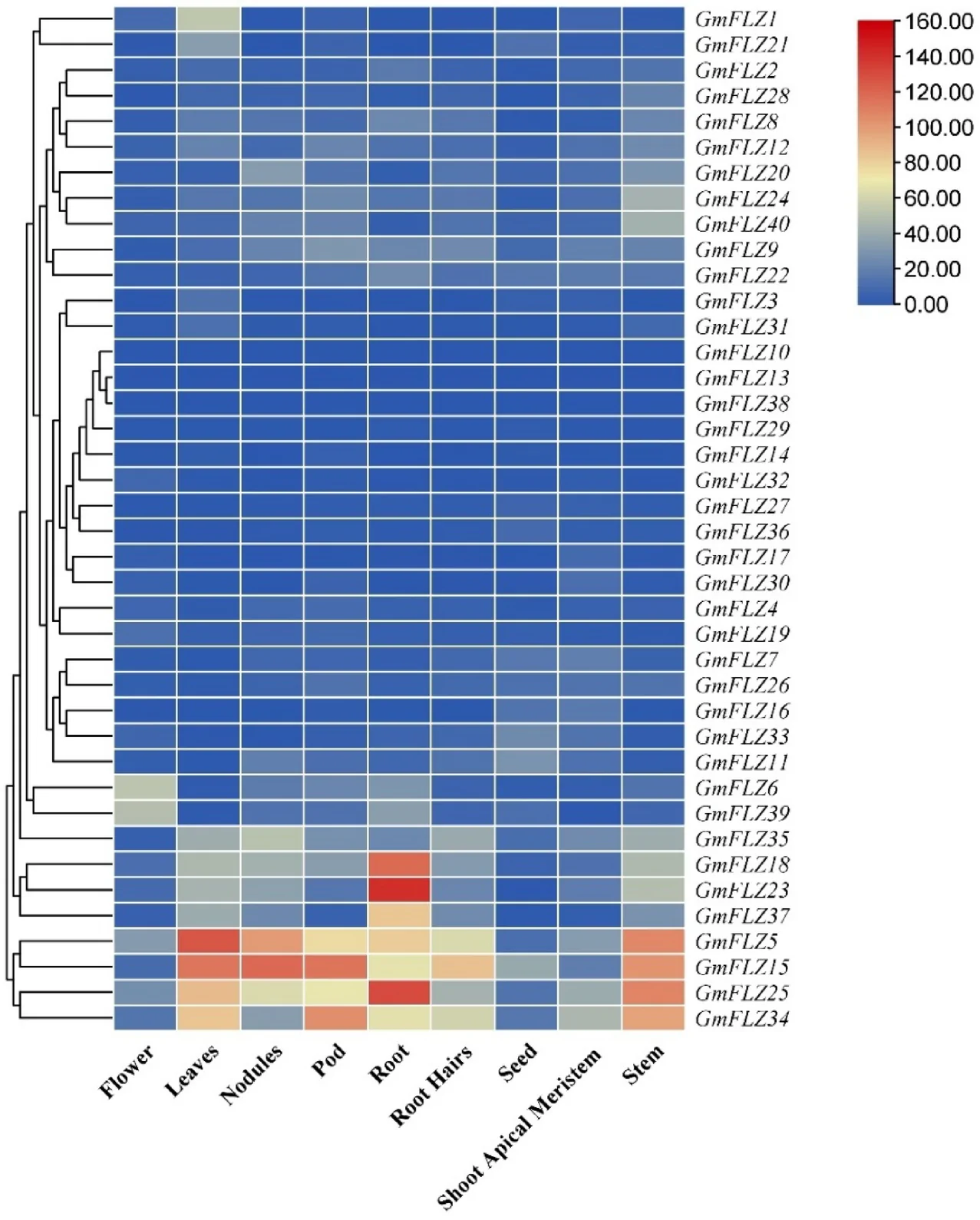
Heat map of tissue-specific expression analysis of FLZ genes from *G. max.* RNA-seq relative-expression transcriptome data from 9 tissues were used to reconstruct the expression patterns of soybean genes.

To characterize more insight into the temporal and spatial expression patterns of the *GmFLZ* gene family, a transcriptomic analysis was performed across nine tissue types, including flower, leaves, nodules, pod, root, root Hairs, seed, shoot apical meristem, and stem, using available RNA-seq datasets. The RNA-Seq Atlas of the *Glycine max* was searched and downloaded for each soybean *FLZ* member (Table S9) were downloaded from the phytozome v14.0 (Joint Genome Institute, JGI) (https://phytozome-next.jgi.doe.gov/; accessed on 13 February 2026) database and visualized as a hierarchically clustered heatmap (Fig. 6), with color intensity ranging from deep blue to bright red. The predominant blue hue in the heatmap matrix indicates that most *GmFLZ* genes had consistently low transcript levels (FPKM = 0–20) across all examined tissues. In contrast, a phylogenetically cohesive cluster comprising *GmFLZ5*, *GmFLZ15*, *GmFLZ25*, and *GmFLZ34* showed the highest expression levels across the GmFLZ family, with *GmFLZ5* emerging as the most highly expressed member in Leaves, Nodules, and Stem, and showing moderate expression in pod, root, and root hairs. *GmFLZ15* showed high expression in Leaves, Nodules, pods, and Stem (Fig. 7). Moreover, *GmFLZ25* showed preferential upregulation in Roots and stems, whereas *GmFLZ34* showed a more moderate expression pattern across various tissues, including Leaves, pods, roots, and Stems. In addition, three *GmFLZ* genes (*GmFLZ18*, *GmFLZ23*, and *GmFLZ37*) showed markedly high transcript abundance in root tissue alone, with the highest expression observed for *GmFLZ23*. Furthermore, *GmFLZ1* showed moderate expression levels in Leaves, distinguishing them from the largely silent majority of *GmFLZ* family members. Finally, all *GmFLZ* family genes show down-expression patterns in the flower, seed, and shoot apical meristem compared with the other tissues.

### Expression profiling of the *GmFLZ* family under drought and salt stress

**Fig. 8:**
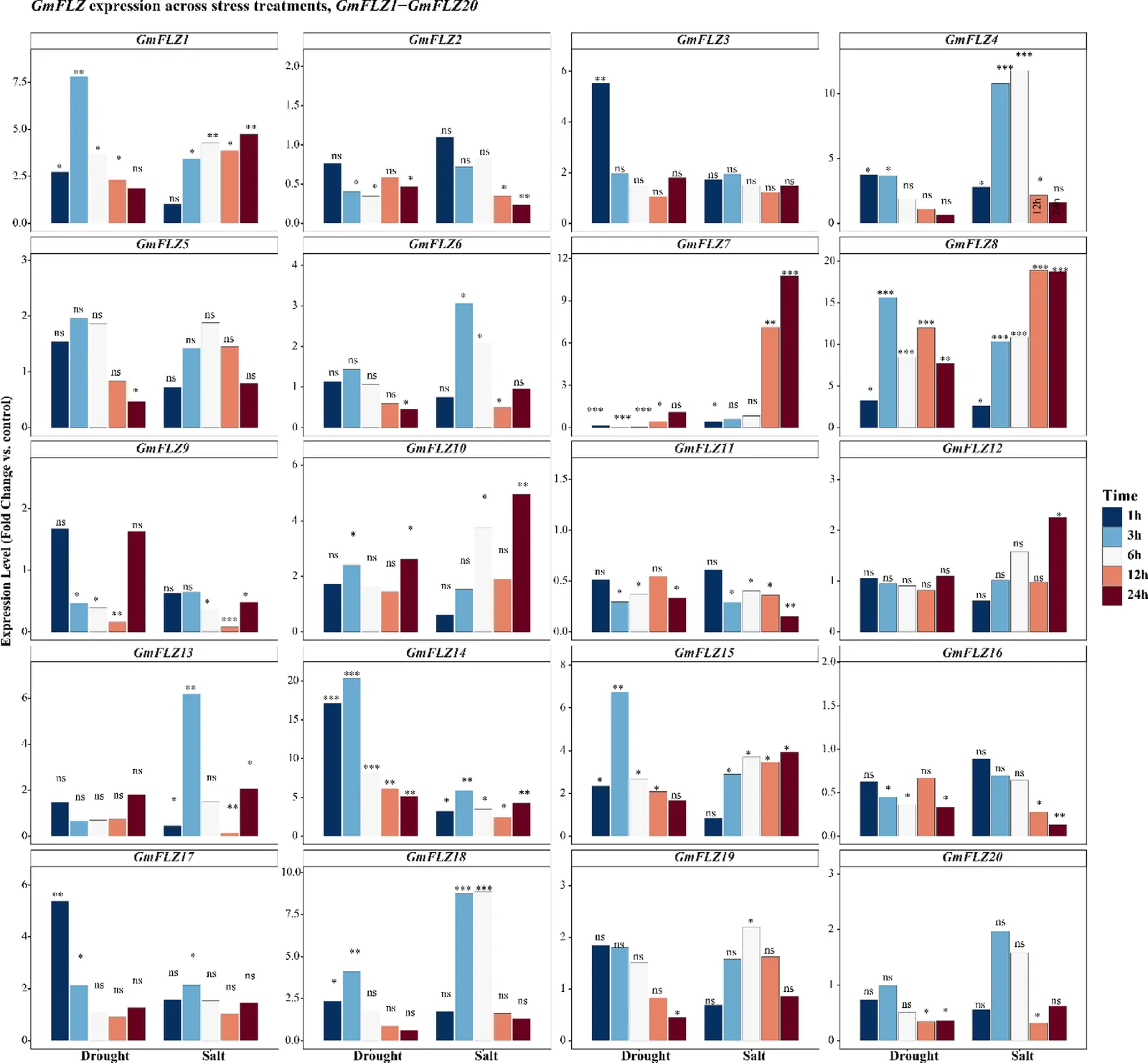
Relative expression of *GmFLZ1* to *GmFLZ20* in Giza 5 soybean seedlings subjected to salt stress (200 mM NaCl) or drought stress (20% PEG-6000), measured by RT-qPCR at 1, 3, 6, 12, and 24 h post-treatment. Expression levels are shown as fold-change relative to the untreated 0 h control, calculated using the 2^-ΔΔCt method with *GmActin11* as the internal reference gene. Bar color denotes sampling time, from blue (1 h) to red (24 h), with the corresponding time point also printed on each bar. Data represent the mean of three biological replicates. Asterisks above bars indicate the magnitude of change relative to the control (ns, log2FC < 1; *, 1 ≤ log2FC < 2; **, 2 ≤ log2FC < 3; ***, log2FC ≥ 3).

**Fig. 9:**
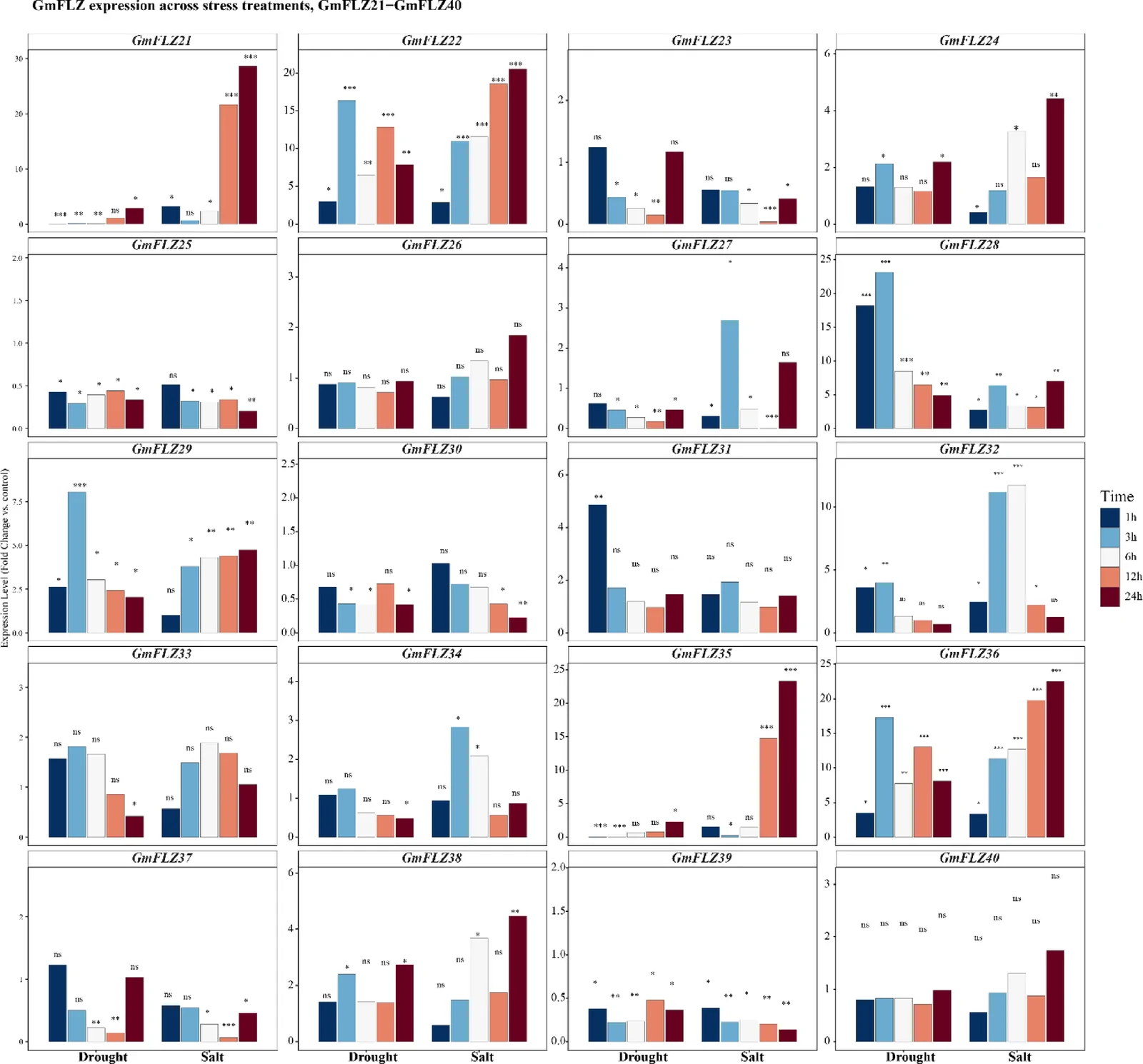
Relative expression of *GmFLZ21* to *GmFLZ40* under the same salt and drought treatments. Expression levels are shown as fold-change relative to the untreated 0 h control, calculated using the 2^-ΔΔCt method with *GmActin11* as the internal reference gene. Bar color denotes sampling time, from blue (1 h) to red (24 h), with the corresponding time point also printed on each bar. Data represent the mean of three biological replicates. Asterisks above bars indicate the magnitude of change relative to the control (ns, log2FC < 1; *, 1 ≤ log2FC < 2; **, 2 ≤ log2FC < 3; ***, log2FC ≥ 3).

Of the 40 *GmFLZ* genes examined, 36 showed at least a twofold change in expression relative to control at one or more time points under drought stress, and 34 did so under salt stress, indicating that most of the family responds to both treatments rather than remaining transcriptionally static (Figs. 8 and 9).

The two stresses differed in how these responsive genes behaved. Under drought, 19 genes were upregulated, and 19 were downregulated at some point in the time course, with two genes showing both directions at different time points, a roughly even split between induction and repression. Under salt stress, the balance shifted toward induction: 24 genes were upregulated and 16 downregulated, with 6 genes showing both directions, suggesting that salt stress more often drives *GmFLZ* expression upward rather than suppressing it. Timing also differed between the two stresses. Under drought, the number of responsive genes was already substantial by 1 h (19 genes) and peaked at 3 h (27 genes), before declining toward 12 h (16 genes) and rising again by 24 h (24 genes), consistent with an early transcriptional response that partially subsides before a later, possibly secondary, wave of change. Under salt, fewer genes responded at 1 h (13 genes), with the number climbing steadily to a plateau at 6–24 h (25, 26, and 25 genes, respectively), indicating a more gradual onset of the salt-responsive transcriptional program compared with drought.

Among individual genes, the strongest responses were recorded for *GmFLZ21* (28.6-fold induction at 24 h under salt), *GmFLZ35* (23.3-fold at 24 h under salt), and *GmFLZ28* (23.1-fold at 3 h under drought), with *GmFLZ36*, *GmFLZ22*, *GmFLZ14*, and *GmFLZ8* also among the most strongly induced genes under one or both stresses. *GmFLZ21* and *GmFLZ35* were of particular note for showing near-complete transcript loss at an early time point (1 h and 3 h, respectively) followed by strong induction later in the time course, a biphasic pattern that, if confirmed once replicate-level statistical testing is available, would suggest tightly time-dependent regulation rather than a simple, continuous response. Most of the responsive genes overlapped between the two stresses: 32 genes crossed the twofold threshold under both drought and salt at some point in the time course, while only four genes responded exclusively to drought and two exclusively to salt. This overlap suggests that the stress-responsive behavior of the *GmFLZ* family is largely shared between these two treatments rather than divided into distinct drought-specific and salt-specific subsets.

### *GmFLZ*-transcription factor co-expression network

**Fig. 10:**
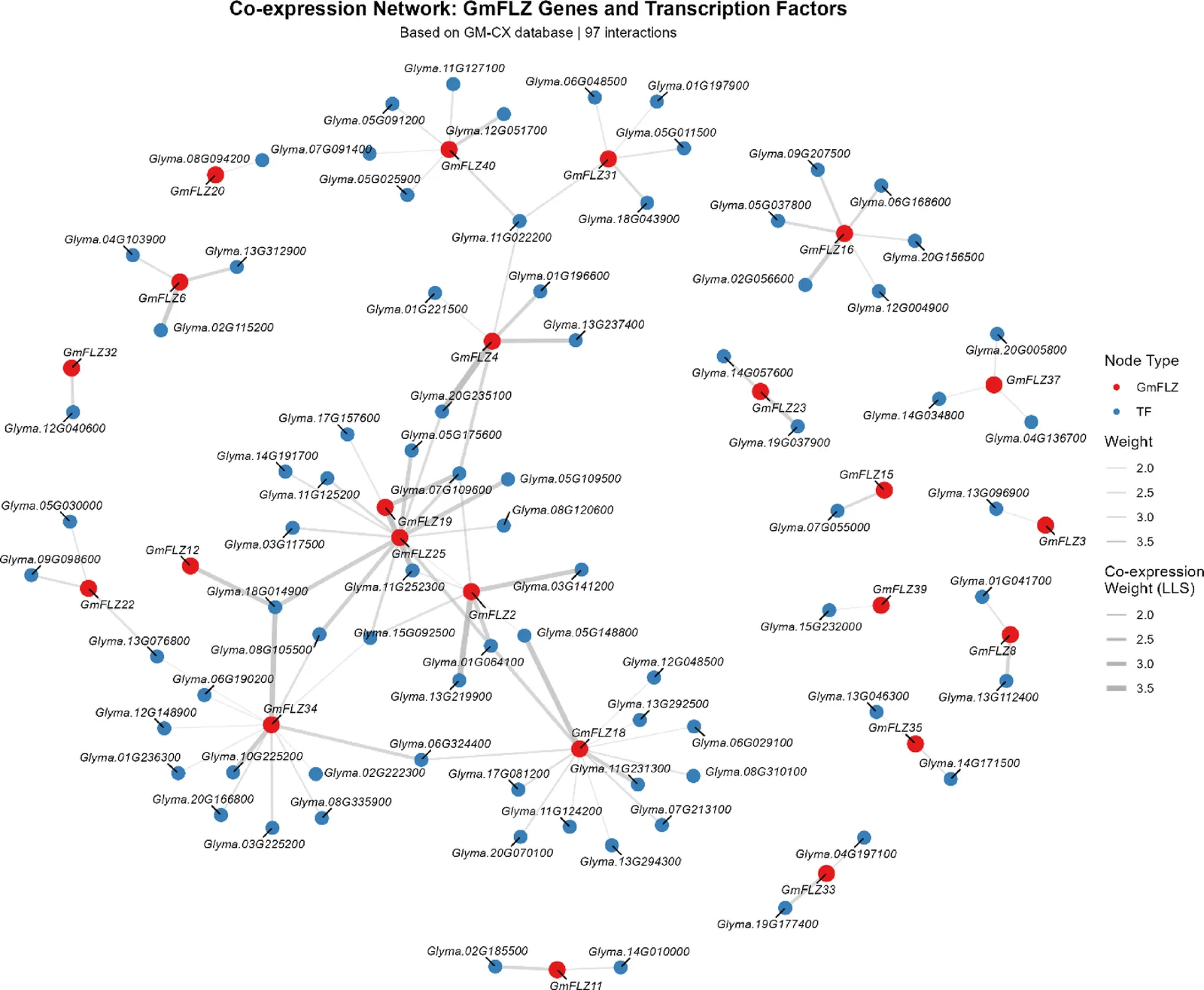
Co-expression network of *GmFLZ* genes and transcription factors based on the GM-CX database. Co-expression network linking members of the *GmFLZ* gene family (red nodes) to putative transcription factor partners (blue nodes), constructed from the GM-CX soybean co-expression database. Edge width and shading represent the co-expression weight (log-likelihood score, LLS); only *GmFLZ*-transcription factor interactions are shown. Gene names are shown in italics.

Querying the GM-CX co-expression database identified 97 co-expression interactions linking members of the *GmFLZ* family to putative transcription factor partners, with co-expression weights (LLS) ranging from 1.82 to 3.76 (mean 2.42) (Fig. 10). Of the 40 *GmFLZ* genes, 23 (57.5%) had at least one qualifying TF co-expression partner, connecting collectively to 79 distinct transcription factors. Connectivity was not evenly distributed across the family. Three genes accounted for a disproportionate share of the network: *GmFLZ25* (15 TF partners), *GmFLZ18* (13), and *GmFLZ34* (13), which together contributed 41 of the 97 interactions (42%), while most other connected genes had considerably fewer partners, *GmFLZ2*, *GmFLZ4*, *GmFLZ16*, and *GmFLZ40* each linked to 6 TFs, and the remaining connected genes had five or fewer. This pattern suggests that *GmFLZ25*, *GmFLZ18*, and *GmFLZ34* may occupy more central positions within the broader transcriptional regulatory network than other family members. Most transcription factors in the network were associated with a single *GmFLZ* gene, but 11 TFs were shared among two or more family members, suggesting potential common upstream regulators. The most broadly connected of these, Glyma.07G109600, co-expressed with four different *GmFLZ* genes, while five additional TFs (*Glyma.11G022200*,* Glyma.01G064100*,* Glyma.18G014900*,* Glyma.11G252300*, and *Glyma.15G092500*) each connected to three. The strongest individual interaction by weight was between *GmFLZ4* and Glyma.20G235100 (LLS = 3.76), followed by *GmFLZ2*-Glyma.13G219900 (LLS = 3.61) and *GmFLZ19*-Glyma.11G252300 (LLS = 3.50).

### Gene Ontology enrichment of the *GmFLZ* co-expression network

**Fig. 11:**
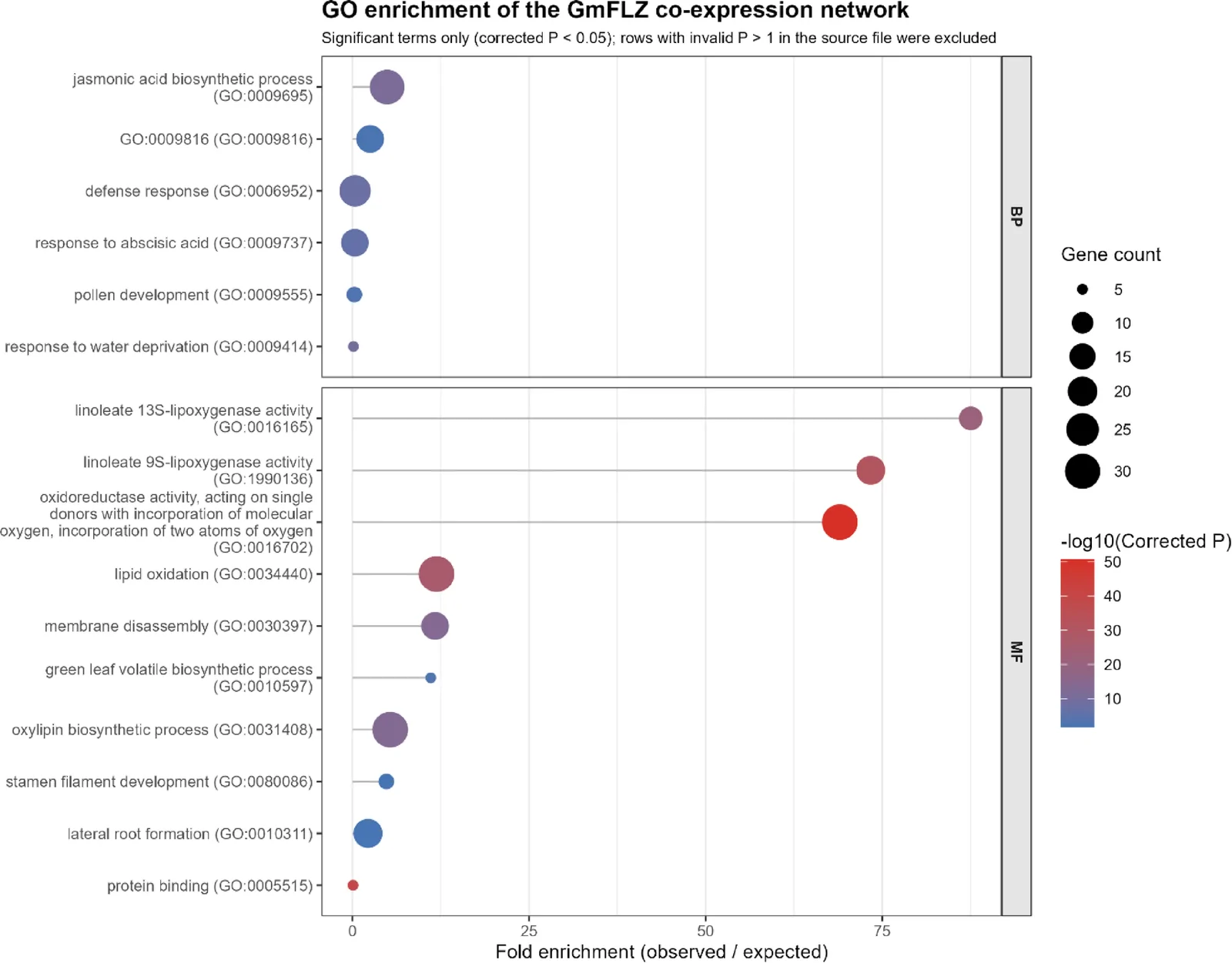
Gene Ontology enrichment of the *GmFLZ* co-expression network. Dot plot of significantly enriched GO terms (corrected *P* < 0.05) among genes co-expressed with the *GmFLZ* family, as identified in the GM-CX database. Terms are grouped by category, biological process (BP) and molecular function (MF), with dot size representing the number of genes annotated to each term and color representing statistical significance (-log10 corrected P). Fold enrichment reflects the ratio of observed to expected gene counts under each term.

GO enrichment analysis of the *GmFLZ* co-expression partner gene set identified 16 significantly enriched terms (corrected *P* < 0.05) among the 29 categories tested, spanning both biological process (BP) and molecular function (MF) domains (Fig. 11). The molecular function category showed the strongest and most consistent enrichment signal, dominated by lipid oxidation and lipoxygenase-related activity. Linoleate 13 S-lipoxygenase activity was enriched 87.6-fold over expectation (corrected *P* = 1.56 × 10^-21), linoleate 9 S-lipoxygenase activity 73.4-fold (*P* = 6.69 × 10^-32), and the broader oxidoreductase activity category (acting on single donors with incorporation of molecular oxygen) 69.0-fold (*P* = 2.20 × 10^-51), the single most significant term in the dataset. Related terms, lipid oxidation (11.9-fold), membrane disassembly (11.7-fold), oxylipin biosynthetic process (5.3-fold), and green leaf volatile biosynthetic process (11.1-fold), were also significantly overrepresented. By contrast, protein binding was strongly underrepresented (0.07-fold, *P* = 3.50 × 10^-40), indicating that the co-expressed gene set is depleted for generic protein-interaction annotations relative to the genome-wide background.

Within biological process terms, the jasmonic acid biosynthetic process was significantly overrepresented (4.9-fold, *P* = 2.60 × 10^-12), consistent with the lipoxygenase and oxylipin signal seen in the molecular function category, since the lipoxygenase pathway feeds directly into jasmonate biosynthesis. In contrast, several general stress- and development-related terms were significantly underrepresented: response to water deprivation (0.13-fold), defense response (0.36-fold), response to abscisic acid (0.33-fold), and pollen development (0.26-fold). An additional overrepresented but functionally unannotated term (GO:0009816, 2.5-fold) was also significant. Taken together, the enriched terms point to a co-expression neighborhood centered on lipoxygenase activity and jasmonic acid biosynthesis, rather than on the broader abiotic-stress or general defense-response categories that were, notably, underrepresented in this same gene set.

## Discussion

### Conservation and molecular evolution of the GmFLZ family

The FLZ protein family has been implicated in abiotic and biotic stress responses, as well as in plant growth and development^34,35^. Most existing knowledge of FLZ genes derives from studies in Arabidopsis^35,36^, rice^9^, wheat^7^, maize^4^, tomato^18^, and cotton^19^, and the functions of many family members remain uncharacterized. To our knowledge, no genome-wide analysis of the FLZ family in soybean has previously been reported^1,37^. Here, we characterized 40 *GmFLZ* proteins in soybean, including their physicochemical properties, phylogenetic relationships, gene structure and motif composition, cis-acting elements, synteny and collinearity, predicted subcellular localization, and tissue-specific expression patterns.

Structural and physicochemical analyses indicated that *GmFLZ* proteins share a conserved FLZ domain but vary considerably in length (105–443 aa), a pattern broadly consistent with the functional divergence reported for FLZ proteins in other plant species^3,19,38^. Using an instability index threshold of 40^39^, we found that 39 of the 40 *GmFLZ* proteins are predicted to be unstable, consistent with the properties generally expected of regulatory proteins. This predicted structural flexibility is characteristic of intrinsically disordered proteins (IDPs), which typically engage binding partners through coupled folding-upon-binding mechanisms^40,41^; this observation is compatible with, though does not directly establish, a role for *GmFLZ* proteins as regulatory scaffolds in stress adaptation and growth regulation. Approximately one-third of *GmFLZ* members had a high aliphatic index (> 70), a property associated with enhanced thermostability in other plant proteins, including in *A. thaliana*^42^. Subcellular localization was predicted in the nucleus for 38 of the 40 *GmFLZ* proteins, with only two members (*GmFLZ13*, *GmFLZ38*) predicted to be extracellular. This pattern aligns with the proposed role of FLZ proteins as regulatory scaffolds that interact with SnRK1-mediated energy signaling and nuclear transcriptional regulators 2^,^45, and is consistent with prior findings in *A. thaliana*, where AtFLZ proteins are predominantly nuclear-localized and implicated in stress signaling^6^.

The uneven distribution of the 40 *GmFLZ* genes across 18 of the 20 soybean chromosomes, with no members detected on chromosomes 8 and 12, resembles patterns reported for other multigene families in polyploid genomes and is generally attributed to preferential gene retention following polyploidization^3,43^. The concentration of four genes on chromosome 17 (*GmFLZ33*, *GmFLZ34*, *GmFLZ35*, *GmFLZ36*) may reflect a local retention hotspot, possibly related to chromatin accessibility or proximity to regulatory elements favoring retention of duplicated copies. Seventeen *GmFLZ* genes were located within the first 10 Mb of their respective chromosomes, a pattern consistent with the tendency of gene-dense regions in the soybean genome to lie near centromeric zones, where WGD-derived segmental duplicates tend to be preferentially retained^44^.

Phylogenetic analysis of the 87 FLZ proteins (40 *GmFLZs*, 18 *AtFLZs*, and 29 *OsFLZs*) resolved four major clades, with *GmFLZ* members distributed across all four alongside their Arabidopsis and rice counterparts, suggesting that FLZ family diversification predates the monocot–dicot split. The relative enrichment of cluster 1 with rice FLZ genes may reflect lineage-specific expansion within monocots, a pattern also reported for other stress-responsive gene families in Poaceae, such as maize^4^. The expansion of the FLZ family in soybean, reflected in the presence of multiple *GmFLZ* members across all major clades, is consistent with gene duplication followed by retention; whether this expansion confers a specific adaptive advantage, rather than simply broadening the functional repertoire available for environmental and developmental responses, remains to be tested.

### Subcellular localization and functional implications

The predicted chloroplast localization of *GmFLZ17*, *GmFLZ26*, *GmFLZ28*, *GmFLZ29*, and *GmFLZ30* is notable, as chloroplast-targeted FLZ proteins have not, to our knowledge, been characterized in detail in any plant species; this may point to a role in protecting the photosynthetic apparatus or in chloroplast-specific stress responses, although this remains speculative without direct evidence. Similarly, the predicted moderate mitochondrial localization of *GmFLZ4*, *GmFLZ19*, and *GmFLZ39* could indicate roles in mitochondrial protein regulation or respiratory-chain protection, though this warrants targeted experimental confirmation. The predominantly nuclear localization predicted for most *GmFLZ* proteins is consistent with a role in ABA-mediated transcriptional regulation and stress-adaptive gene expression, in line with prior work on Arabidopsis FLZ proteins, where nuclear-localized members have been reported to influence SnRK1 kinase activity^6^.

### Gene structure and functional diversification

Consistent with findings in Arabidopsis, maize, and tomato FLZ families^4,5,18^, we identified ten conserved motifs, with Motifs 1 and 2 present in all 40 *GmFLZ* members, suggesting a conserved role for these motifs in maintaining the structural integrity of the zf-FLZ domain. The stepwise acquisition of additional motifs in structurally more complex subgroups may reflect progressive functional elaboration through motif recruitment during evolutionary divergence, a mechanism reported for other plant regulatory protein families^45^. *GmFLZ1*, which forms a singleton within its cluster and shows an atypical motif composition, may represent a neofunctionalized gene with distinct regulatory properties, though this interpretation would benefit from functional validation. The relatively narrow E-value range for the zf-FLZ domain across the family (1.1 × 10⁻²¹ to 9.8 × 10⁻³¹) indicates a high degree of sequence conservation, consistent with an important functional role for this domain.

### Gene duplication, synteny analysis, and evolutionary origin

Segmental duplication associated with whole-genome duplication (WGD) is a well-documented contributor to gene-copy expansion in the soybean genome. Consistent with this, the 304 paralogous pairs identified here 290 segmental and 14 tandem duplicates suggest that segmental duplication is the primary mechanism underlying *GmFLZ* family expansion^44^, paralleling findings for FLZ families in *Z. mays*^4^ and *Brassica sp.*^3^. The predominance of purifying selection (Ka/Ks < 1 in 97.9% of pairs; mean Ka/Ks = 0.30) suggests that the zf-FLZ domain is under functional constraint, while six paralogous pairs with Ka/Ks > 1, most notably *GmFLZ13*/*GmFLZ14* (Ka/Ks = 2.63) may indicate localized positive selection consistent with neofunctionalization.

Divergence times among paralogous pairs spanned a wide range (2.9–263.9 Mya; mean = 135.5 Mya), with most divergence events occurring during the legume-specific WGD period. The conservation of syntenic links between *GmFLZ* genes and their counterparts in *A. thaliana*, *O. sativa*, and *Z. mays* is consistent with an ancient origin for the FLZ family, predating the monocot–dicot split (~ 150–250 Mya). The correspondence between multiple soybean chromosomal segments and individual Arabidopsis regions is compatible with the paleopolyploid structure of the soybean genome and resembles synteny patterns reported for other gene families in *G. max*^44^. The persistence of synteny over such evolutionary distances suggests that FLZ loci may be subject to constraints on chromosomal rearrangement, potentially reflecting co-localization with conserved regulatory elements, though this remains to be tested directly.

### Cis-acting elements and regulatory diversity

ABA-mediated signaling is an important regulator of plant responses to drought and salinity, and FLZ-domain proteins have been reported to act as positive regulators of ABA signaling in Arabidopsis; against this background, the prevalence of ABA-responsive elements across most *GmFLZ* promoters is notable^44^. The co-occurrence of MeJA-responsive, salicylic acid-responsive, drought-inducible, and low-temperature-responsive elements within a subset of promoters is consistent with regulatory crosstalk among hormone-mediated stress pathways, and suggests that a substantial proportion of *GmFLZ* genes may be integrated into more than one stress-signaling network^46^.

### Tissue-specific expression and developmental roles

The transcriptional heterogeneity observed among *GmFLZ* members is consistent with sub-functionalization following gene duplication: a distinct subset shows tissue-preferential upregulation, while most members show consistently low expression. A comparable pattern has been reported for other soybean gene families, including WRKY, MYB, and bZIP transcription factors, in which only a small fraction of paralogs are constitutively expressed while most are induced by specific biotic or abiotic stimuli^44,46^. Within the *GmFLZ* family, *GmFLZ5* showed elevated expression in leaves, nodules, and stems, and *GmFLZ15*, *GmFLZ25*, and *GmFLZ34* showed elevated expression in overlapping tissue sets; together, these four genes may constitute a functionally distinct subgroup associated with metabolic activity in vegetative tissues. However, because most *GmFLZ* members showed comparatively low expression in reproductive organs and the shoot apical meristem, broader claims about a role in soybean development should be treated as provisional pending functional validation. *GmFLZ18*, *GmFLZ23*, and *GmFLZ37* showed preferential expression in root tissue and may be reasonable priorities for functional characterization, potentially in relation to nodule development or rhizosphere-associated stress responses, though this remains to be tested directly.

## Conclusion

This study identified 40 members of the *GmFLZ* gene family distributed across 18 soybean chromosomes, representing, to our knowledge, one of the first genome-wide identifications and characterizations of this family in *Glycine max*. Phylogenetic analysis of GmFLZ proteins alongside Arabidopsis and rice FLZ orthologs resolved four major clades, consistent with an evolutionary origin predating the monocot–dicot divergence. Gene duplication analysis identified 304 paralogous pairs, predominantly arising from segmental duplication under purifying selection (97.9% of pairs), and structural analysis identified ten conserved motifs, with Motifs 1 and 2 present in all family members. *GmFLZ18*, *GmFLZ23*, and *GmFLZ37* showed root-preferential expression, and *GmFLZ5*, *GmFLZ15*, *GmFLZ25*, and *GmFLZ34* showed comparatively high expression across vegetative tissues, based on tissue-specific expression profiling. RT-qPCR profiling under drought and salt stress further indicated that most *GmFLZ* genes are transcriptionally responsive to these treatments (36 and 34 of the 40 genes showed at least a two-fold change under drought and salt stress, respectively), providing experimental support for the role of this family in abiotic stress responses. Together, these findings provide an evolutionary and functional framework for the *GmFLZ* family in soybean and identify candidate genes that may be prioritized for functional validation in efforts to improve stress tolerance.

## Supplementary Information

Below is the link to the electronic supplementary material.

Supplementary Material 1

## Data Availability

The soybean genome and proteome data used in this study were downloaded from Phytozome v14.0 (https://phytozome-next.jgi.doe.gov/). Arabidopsis thaliana (TAIR10) and Oryza sativa (RAP-DB MSU v7.0) sequences are available at their respective public repositories. All supplementary tables (Tables S1–S8), primer sequences. Raw numerical data supporting the figures are available from the corresponding author upon reasonable request. No previously unpublished sequencing datasets were generated in this study.

## Abbreviations

ABA: Abscisic acid
BLAST: Basic local alignment search tool
CDS: Coding sequence
CRediT: Contributor roles taxonomy
DUF581: Domain of unknown function 581
FPKM: Fragments per kilobase of transcript per million mapped reads
FLZ: FCS-like zinc finger
GmFLZ: Glycine max FCS-like zinc finger
GRAVY: Grand average of hydropathicity
HMM: Hidden Markov model
IDPs: Intrinsically disordered proteins
Ka: Non-synonymous substitution rate
Ks: Synonymous substitution rate
MeJA: Methyl jasmonate
MIQE: Minimum information for publication of quantitative real-time PCR experiments
MWS: Molecular weight
MW: Molecular weight
Mya: Million years ago
NJ: Neighbour-joining
pI: Isoelectric point
PEG: Polyethylene glycol
PPI: Protein–protein interaction
qRT-PCR: Quantitative reverse transcription PCR
SnRK1: Sucrose non-fermenting 1-related protein kinase 1
SnRK2/3: Sucrose non-fermenting 1-related protein kinase 2/3
WGD: Whole-genome duplication
WoLF PSORT: WoLF protein sorting prediction
zf-FLZ: Zinc finger FLZ domain (Pfam: PF04570)
